# Estimated average glucose: A new term in diabetes control

**DOI:** 10.4103/0256-4947.59375

**Published:** 2010

**Authors:** Jean G. Dib

**Affiliations:** From the Pharmacy Services Division, SAMSO, Dahran, Saudi Arabia

**To the Editor:** Glycated or glycosylated hemoglobin (HbA_1c_) levels have been used in planning and assessing the management of diabetic patients for the past couple of decades. Clinical trials have established the correlation between HbA_1c_ and the development of diabetes complications and patient outcomes.^1^,^2^ HbA_1c_ results are expressed as the percentage of hemoglobin that is glycated and reflects the average blood glucose control over a period of approximately three months. In contrast, blood glucose levels are expressed in milligrams per deciliter and are used for daily monitoring by the patient and healthcare professionals. The discrepancy between HbA_1c_ and blood glucose level units has been problematic and has created some confusion among patients. To reduce this confusion, researchers have determined and reported a linear correlation between HbA_1c_ and self-monitored glucose levels obtained by frequent fingerstick capillary glucose testing and continuous glucose monitoring. A mathematical relationship between the average glucose level over the preceding three months and levels of HbA_1c_ has been established,^3^ resulting in the translation of HbA_1c_ results into estimated average glucose (eAG). This approach was adopted by the American Diabetes Association (ADA).^4^,^5^

The estimated average glucose (eAG) converts the diabetic patient's HbA_1c_ percentage point into an average blood glucose level in the units of measure seen by the patient on glucose meters for daily self-monitoring (mg/dL). Similar to HbA_1c_, eAG evaluates a patient's overall success at controlling glucose levels and helps patients understand the monitoring of their long-term treatment. The relationship and association between HbA_1c_ and eAG has been supported by several studies with the largest being published by the HbA_1c_-Derived Average Glucose (ADBG) Study Group.^3^ The ADAG linear regression formula that is used to calculate the eAG from A_1c_ results is: “28.7×A_1c_-46.7”, and therefore, every one percent increase in HbA_1c_ is approximately equivalent to an increase of 29 mg/dL in eAG (Table 1).^4^,^6^

**Table 1:** The relationship between glycated hemoglobin and estimated average glucose.

A_1c_ (%)	eAG (mg/dL)
6	126
6.5	140
7	154
7.5	169
8	183
8.5	197
9	212
9.5	226
10	240

A_1c_: Glycated hemoglobin; eAG: estimated average glucose; Formula utilized: eAG=28.7×A_1c_-46.7

As a clinical pharmacist practising in an ambulatory care setting, I have noted that patients find it difficult to explain and relate the HbA_1c_ percentage of hemoglobin that is glycated to glucose measurements that they encounter through home or lab glucose monitoring (“numbers are not matching”). Since the introduction of eAG, I have used it to describe three-month blood glucose control in an easily comprehensible way that can be correlated to their home glucose testing. As a first impression, patients appear to quickly accept and welcome eAG. With eAG, patients will have a better understanding of glucose monitoring and can now correlate the results of HbA_1c_ to eAG. Due to the added advantage that eAG brings to patient care, we expect that future consensus guidelines will recommend that laboratories report calculated eAG levels along with A_1c_ values.
